# Upgrading the HIV vaccine paradigm: accounting for host-pathogen diversity through geotargeting-based vaccine design

**DOI:** 10.3389/fcimb.2026.1927865

**Published:** 2026-10-16

**Authors:** Luciano Lunardi, Alexandra de Azevedo Rocha, Ana Carolina Alves de Oliveira, Iuri Marques de Oliveira, Angelica Cerveira De Baumont, Dinler Amaral Antunes, Gustavo Fioravanti Vieira

**Affiliations:** 1 Grupo Hospitalar Conceicao, Porto Alegre, Brazil; 2 Universidade Federal do Rio Grande do Sul, Porto Alegre, Brazil; 3 PPG Biosaúde Ulbra, Universidade Luterana do Brasil, Canoas, Brazil; 4 Responsabilidade Social, Hospital Moinhos de Vento, Porto Alegre, Brazil; 5 Department of Biology and Biochemistry, University of Houston, Houston, TX, United States; 6 Universidade La Salle Canoas, Canoas, Brazil

**Keywords:** elite controllers (EC), HIV, HLA class I, immunodominant epitopes, immunoinformatics, vaccine design

## Abstract

**Introduction:**

Effective HIV vaccine development, with satisfactory levels of response and coverage, remains an elusive goal in immunology. In this context, considerations of host-pathogen interactions from a geotargeting perspective could enhance disease control efforts. Current HIV vaccine strategies increasingly seek to address viral diversity, but the combined effects of regional viral variation and host HLA diversity on antigen processing and presentation remain insufficiently considered. Here, we evaluated whether integrating these host–pathogen variables could support geographically informed prioritization of HIV vaccine targets.

**Methods:**

Using NetMHCpan 4.1, NetChop 3.1, and the IEDB antigen-processing prediction tool, we evaluated antigen-processing and presentation steps for HIV epitopes associated with viral control. We recovered Human Leukocyte Antigen (HLA) alleles associated with HIV control and their frequencies, and the protein sequences for the most common HIV subtypes in different African countries. We created maps linking both sets of information for each specific region. We then predicted the impact of mutations associated with different HIV strain sequences on proteasome processing score, TAP transportation and HLA binding affinity for the alleles in each host population.

**Results:**

The generated maps provide a landscape of the impact of genetic diversity on T cell responses for each examined epitope target. TW10 epitope variants in the main HIV subtypes showed consistent HLA binding, proteasome cleavage and processing rankings across the continent. Conversely, TI8 variants carrying I8T or I8R substitutions lost predicted HLA-B*51:01 binding and showed marked deterioration in the combined processing ranking, from 10th for the reference epitope to positions as low as 97th among the analyzed variants.

**Discussion:**

The adoption of uniform immunotherapy strategies based solely on reference HIV epitopes may be an inefficient approach. Certain epitopes may be suitable vaccine targets for specific locations, when matching a prevalent HIV strain with a host population with high prevalence of HLA alleles that can effectively display the vaccine’s epitopes. However, HLA matching is not enough if the HIV strains in the region have upstream or downstream mutations that prevent the proper processing and presentation of the targeted viral epitope. Based on this complex host–pathogen interplay, we propose a geotargeting approach to vaccine design, considering customized targets for specific geographical locations and host populations. By incorporating regional viral and HLA diversity into target selection, this strategy may increase the likelihood of developing more effective HIV vaccines, although the resulting predictions will require experimental validation.

## 1 Introduction

Despite exciting progress over the years, HIV continues to be a challenging target for vaccine development. Many different factors contribute to this problem, including (i) the difficulty of inducing broadly neutralizing antibody responses by vaccination (Burton and Hangartner, 2016), (ii) viral escape mechanisms (Larijani et al., 2019), (iii) the persistence of infected latent cells that are not eliminated by the host’s immunity or long-term antiretroviral therapy (Margolis et al., 2020), (iv) the extensive genetic diversity of HIV (Ng’uni et al., 2020), and (v) host genetic heterogeneity, particularly HLA variation associated with differences in HIV disease outcome (Crux and Elahi, 2017). Among those factors, viral diversity is a major challenge. HIV-1 has four main groups (M, N, O and P) and many subtypes and circulating recombinant forms (CRFs) (Ng’uni et al., 2020) (e.g., CRF02_AG, found in Africa). Interestingly, co-infections by more than one viral variant are common, increasing the difficulty to create a single and effective vaccine for all patient populations. Preventive HIV vaccines showed little protection until now, with around 30% efficacy in one clinical trial in Thailand (Rerks-Ngarm et al., 2009), and failure to prevent HIV infection when using an adenovirus containing mosaic antigens from different HIV strains in Africa (Gray et al., 2024). Mosaic immunogens represent an important strategy to address HIV diversity by computationally combining viral sequences to maximize the coverage of potential T-cell epitopes across genetically diverse HIV variants (Fischer et al., 2007). Consensus-based approaches similarly attempt to reduce the impact of viral diversity by generating representative centralized immunogens (Hauser et al., 2021). Building on the previously proposed geotargeting concept (Lunardi et al., 2021), the present study does not introduce geographic tailoring per se, but operationalizes it as an integrative computational framework that jointly considers the circulating HIV variants, the HLA composition of the host population, and the preservation of multiple steps of antigen processing and presentation. Thus, rather than maximizing global epitope breadth, this framework aims to prioritize regionally appropriate targets whose processing and presentation are predicted to be preserved in a specific host–pathogen context. This concept is also aligned with the broader development of precision vaccinology, which seeks to incorporate host genetic and immunological heterogeneity into vaccine design and deployment (Rahman et al., 2024).

An ideal viral vaccine should include the stimulation of humoral and cellular immunity, especially if the goal is also the elimination of infected cells (Larijani et al., 2019). In this context, cytotoxic T lymphocytes (CTLs) are very important to control HIV infection, especially when targeting key viral antigens such as Gag protein epitopes (i.e., Gag-derived peptides that can be recognized by the immune system). Gag-derived targets are particularly relevant because escape mutations in functionally constrained p24 Gag regions can impose substantial viral fitness costs (Martinez-Picado et al., 2006). Thus, conserved HIV regions such as Gag-derived epitopes represent better targets for wide spectrum vaccine design than other protein antigens presenting higher sequence diversity, as the envelope proteins (Collins et al., 2020). However, narrow focus on a single (or a few) “conserved” epitopes, as pursued in many traditional vaccine designs, often fails to induce responses against the full range of HIV variants, and is more prone to viral escape. In this sense, new vaccine strategies are required, especially those including the production of intracellular viral antigens to generate both cellular and humoral responses. Recently, the higher efficacy of mRNA vaccines, as demonstrated during the SARS-CoV-2 pandemic, opened new avenues for mRNA-based anti-HIV vaccines inducing intracellular production of selected HIV antigenic targets.

Other insights into vaccine development can be drawn from elite controllers (EC), HIV patients with a very low or undetectable viral load and a high CD4 count, in the absence of antiretroviral therapy (Bendenoun et al., 2018). Immunological features of these individuals deserve further investigation, since immune-mediated mechanisms contribute to control of infected cells and maintenance of an undetectable viral load (Gebara et al., 2019). The EC appears to control HIV infection through a cell-mediated immune response, including natural killer cells and virus-specific CD8+ cytotoxic T cells (CTLs) recognizing HIV peptides displayed by Class-I Human Leukocyte Antigen (HLA) molecules (Gebara et al., 2019). The CD8+ CTLs of EC can display enhanced expansion, perforin expression, cytokine production, and antiviral function (Rutishauser and Trautmann, 2022), highlighting the relevance of HLA-restricted cellular responses in these patients. In this context, a successful vaccine design must consider both the expressed HLA alleles in the immunized population, and the circulating HIV strains in the respective location (Collins et al., 2020; Lunardi et al., 2021).

In fact, some HLA alleles have already been associated with HIV control, such as HLA-B*27, HLA-B*51, HLA-B*57, HLA-B*58:01, HLA-A*74:01, and HLA-B*14 (Goulder and Walker, 2012). Again, it is important to understand that this effect is context-specific. For example, a study of Japanese HIV patients found that an 8-mer epitope (TI8, TAFTIPSI) restricted to HLA-B*51:01 is associated with slow disease progression and that CTLs specific for this epitope can inhibit HIV replication *in vitro* (Motozono et al., 2014). On the other hand, a systematic analysis from five established African cohorts associated HLA-B*51:01 with disease progression and higher HIV viral load, highlighting the possibility that the same alleles can determine different outcomes in different ethnic groups (Carlson et al., 2012). More recent large-scale genomic studies further support the central contribution of HLA class I variation to spontaneous HIV control. Multi-ancestry fine-mapping identified amino acid positions within the HLA-B peptide-binding groove as the strongest genetic determinants of HIV-1 set-point viral load, while a subsequent large multiancestry analysis of 9,705 individuals confirmed strong associations between the HLA region and both viral load and spontaneous HIV control (Luo et al., 2021; Vergara et al., 2023). Besides the HLA molecules, other steps of the endogenous peptide presenting pathway can shape the immunopeptidome of HIV-infected patients, including proteasome processing, TAP translocation and trimming by cytosolic aminopeptidases (Blum et al., 2013). Consequently, sequence diversity between HIV variants circulating in different regions can also introduce differences in response between patients, even if these patients share both the HLA allele and the particular HIV-derived epitope targeted by a given vaccine design.

We hypothesized that HIV epitopes previously associated with viral control would retain their potential as geographically targeted vaccine candidates only when locally circulating viral variants preserve the molecular determinants required for antigen processing and HLA presentation in the corresponding host population. Specifically, mutations within the epitope or its flanking regions were expected to reduce candidate suitability when they impaired proteasomal cleavage, HLA binding, or the integrated antigen-processing ranking relative to the reference sequence.

In this context, our study aims to investigate the diversity of HIV subtypes circulating in the African continent, and the impact of protein sequence diversity in antigen processing and presentation, in the context of HLA alleles associated with EC. We also present evidence that allele frequency variation and viral diversity may impair the coverage and efficacy of vaccination strategies that do not consider them. Finally, we leverage immunoinformatic methods and available experimental data to evaluate a geotargeting framework for antiviral target prioritization. We initially focused on the African continent, as proof-of-concept, since it includes some of the countries with the biggest AIDS burden in the world. However, this approach can be applied to any geographical region of interest, considering local viral variants and HLA prevalences. Accordingly, the specific epitope prioritizations and geographic recommendations derived here should be interpreted as Africa-specific and should not be directly extrapolated to other regions, where distinct combinations of HLA allele frequencies and circulating HIV variants would require independent evaluation at the appropriate geographical scale, potentially including national or subnational populations.

## 2 Methods

The complete analytical workflow employed in this study is illustrated in **Figure 1**. The approach integrates regional HLA allele frequencies, circulating HIV-1 subtype sequences, epitope-specific mutations, and predictions of antigen processing and presentation to generate geographically informed maps of potential immune escape. The individual data-retrieval, mapping, and prediction steps are described in detail in the following subsections.

**Figure 1:**
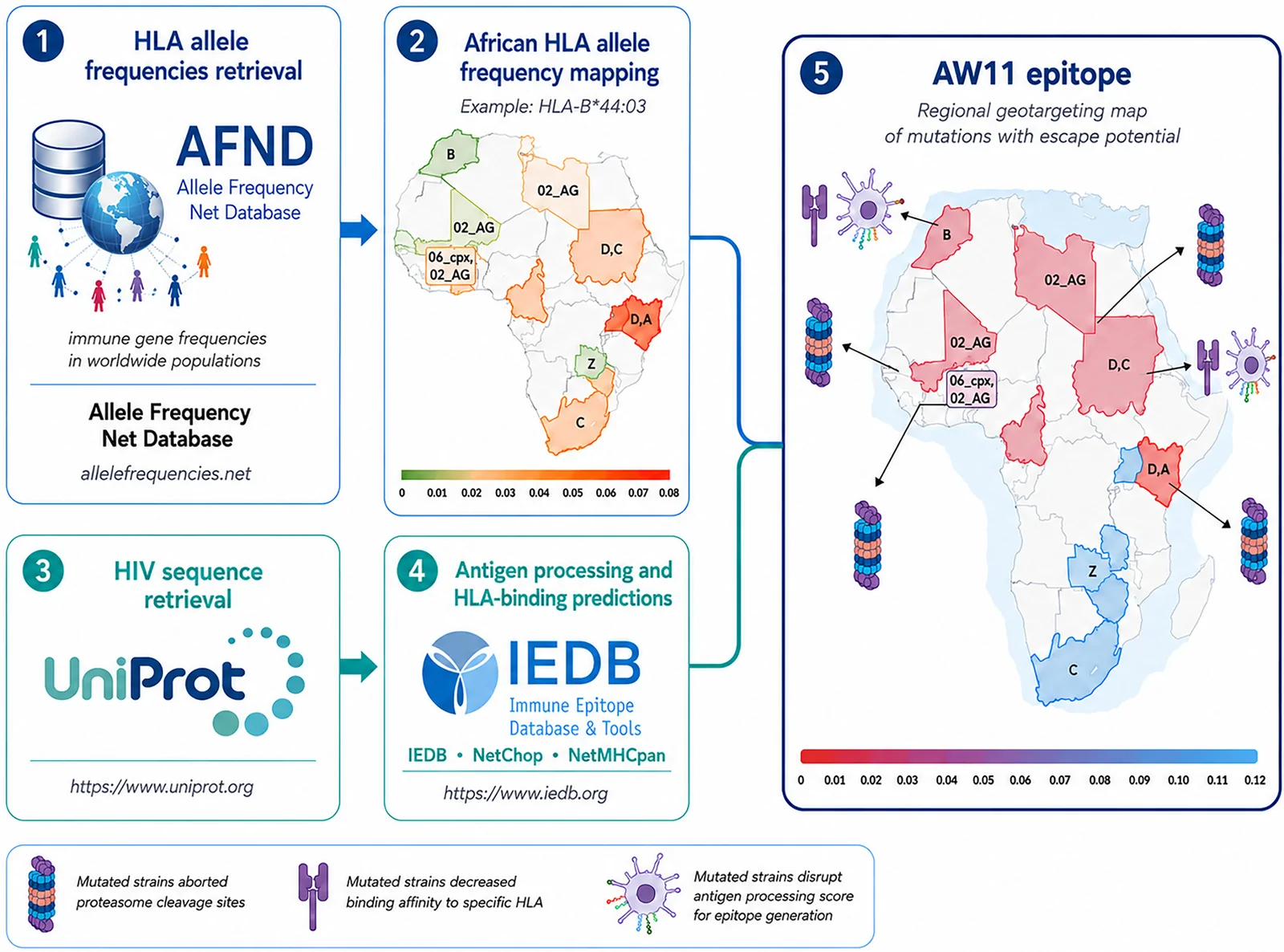
Overview of the immunoinformatics geotargeting workflow used to integrate host HLA diversity and HIV-1 sequence variation. (1) HLA alleles associated with HIV control and their population frequencies were retrieved from the Allele Frequency Net Database. (2) HLA allele frequencies were mapped across African countries and associated with the regional distribution of major HIV-1 subtypes. (3) HIV-1 protein sequences and subtype-specific epitope variants were retrieved from UniProt. (4) The effects of sequence variation on antigen processing and presentation were assessed through predictions of proteasomal cleavage, TAP transport, HLA class I binding, and integrated antigen-processing behavior. (5) The resulting information was integrated into epitope-specific regional maps highlighting geographic settings in which mutations were predicted to abolish proteasomal cleavage, reduce HLA binding affinity, or impair epitope generation. The AW11 epitope is shown as a representative example. Color gradients indicate the frequencies of the relevant HLA alleles, whereas labels within countries indicate the predominant HIV-1 subtype or subtypes.

### 2.1 HLA allele selection and frequency recovering

We conducted an in-depth examination of HLA class I alleles associated with HIV control. The aim of this selection was not to perform a *de novo* survey of all host genetic variants associated with HIV control, but rather to identify well-established HLA–epitope pairs suitable for the subsequent antigen-processing analyses. We therefore prioritized HLA alleles with previously reported associations with spontaneous HIV control and for which specific HIV-derived CD8+ T-cell epitopes had been experimentally characterized (Goulder and Walker, 2012; Motozono et al., 2014). In this study, we included six epitopes derived from the Gag protein: TW10 (TSTLQEQIAW), KF11 (KAFSPEVIPMF), AW11 (AEQATQDVKNW), TL9 (TPQDLNTML), KK10 (KRWIILGLNK), GR11 (GQMVHQAISPR); as well as the TI8 (TAFTIPSI) epitope derived from the reverse transcriptase protein. This panel was intentionally non-exhaustive and was not intended to encompass all HLA alleles associated with HIV control; other relevant alleles, including HLA-B81, HLA-B14, and HLA-B*13, represent important candidates for future expansion of the framework.

We gathered HLA frequency information from the Allele Frequency Net Database (www.allelefrequencies.net), focusing on African populations due to their high HIV prevalence. The selected HLA alleles associated with EC included HLA-B*57:01, B*57:03, B*58:01, B*27:05, B*42:01, B*44:03, and A*74:01 (Goulder and Walker, 2012). Additionally, we included HLA-B*51:01, based on findings supporting its involvement in HIV control (Motozono et al., 2014), while acknowledging its association with poorer disease outcomes in some African cohorts (Carlson et al., 2012).

### 2.2 Distribution of HIV subtypes in Africa and protein recovering

The distribution of HIV-1 subtypes in Africa was determined based on data from the HIV Sequence Database maintained by Los Alamos National Laboratory and available at https://www.hiv.lanl.gov/components/sequence/HIV/geo/geo.comp. Because this database is not derived from a population-based epidemiological sampling framework, the relative frequency of sequences was not interpreted as an estimate of subtype prevalence in the underlying population. Instead, these data were used to characterize the geographic representation of circulating viral diversity available in the database.

HIV-1 protein sequences were retrieved from UniProt using taxonomy-based searches for subtypes A, B, C, D, and CRF02_AG. These viral backgrounds were selected because they represent major subtype and recombinant lineages relevant to the African geographic context evaluated in this proof-of-concept. The sequence dataset used for the analyses had been assembled by September 2021. Rather than selecting a single representative strain for each subtype, sequences containing the analyzed epitope regions were examined to capture subtype-associated variation in the epitope and its surrounding residues relevant to antigen processing. The corresponding UniProt accessions and analyzed sequences are provided in **Supplementary Material 8**.

### 2.3 Map building

The HLA allele frequencies were obtained from www.allelefrequencies.net. When more than one population sample was available for a given country, HLA allele frequencies were aggregated using a sample-size-weighted mean. For each allele, the country-level weighted frequency was calculated as f_country = Σ(f_i_ × n_i_)/Σn_i_, where *fi* represents the allele frequency reported for population sample *i* and *ni* its corresponding sample size. Thus, population samples with larger sample sizes contributed proportionally more to the aggregated estimate. The calculation was weighted exclusively by sample size and did not incorporate national population size or study year. When only one population sample was available for a country, the reported frequency was retained without aggregation. These values should therefore be interpreted as sample-size-weighted estimates derived from the populations available in the database, rather than as nationally representative allele-frequency estimates.

To assess the robustness of country-level HLA frequency estimates to the composition of the available population samples, we performed a leave-one-population-out sensitivity analysis for country–allele combinations represented by more than one population sample. The sample-size-weighted frequency was recalculated after sequentially excluding each population sample, and the resulting minimum and maximum estimates were recorded. Country–allele combinations represented by a single population sample were retained but explicitly identified as estimates for which such sensitivity analysis could not be performed. Sample sizes and the number of contributing population samples are provided in **Supplementary Material 14**.

Correspondence between HLA alleles and each HIV epitope was determined by combining information from the relevant literature and predictions generated using bioinformatics methods. Subsequently, we generated individual maps for each target, illustrating the predominant HIV subtypes and the corresponding HLA allele frequencies associated with each HIV epitope in each country. These informative visuals were crafted using infographics hosted on https://infogram.com. To convey the gradient of allele frequencies, we employed a color spectrum on the maps.

All data aggregation was performed using spreadsheet-based calculations, and no custom Python or R scripts were used. Geographic maps were generated using the built-in visualization tools available on the Infogram platform.

### 2.4 Effect of epitope mutations on the antigen processing pathway

We evaluated the influence of epitope mutations on target recognition and effective immune stimulation through a series of *in silico* predictions for each stage of the antigen processing pathway:

#### 2.4.1 MHC binding affinity

We utilized NetMHCpan 4.1 (https://services.healthtech.dtu.dk/services/NetMHCpan-4.1/) to predict HLA binding affinity (Hoof et al., 2009). Several parameters were included in this assessment including epitope length and %Rank, which represents the ranking of the predicted binding score when compared to a set of random natural peptides. Predicted HLA-binding capacity was interpreted according to the default thresholds implemented in NetMHCpan 4.1. According to the default NetMHCpan 4.1 classification thresholds, peptides with a %Rank < 0.5 were classified as strong binders, those with a %Rank between 0.5 and 2.0 as weak binders, and those with a %Rank ≥ 2.0 as non-binders.

In addition to the alleles for which the epitopes were originally described, we also predicted their binding affinity for other HLA alleles associated with elite controllers (EC), as well as representative alleles for HLA supertypes (HLA-A*02:01, HLA-A*01:01, HLA-A*03:01, HLA-A*24:02, HLA-A*26:01, HLA-B*07:02, HLA-B*08:01, HLA-B*27:05, HLA-B*39:01, HLA-B*40:01, HLA-B*58:01, HLA-B*15:01). For each specific epitope, all variants were submitted to predictions based on the criteria described above.

* For the 11-mer GR11 epitope (GQMVHQAISPR), the three possible overlapping 9-mer peptides were evaluated for binding to HLA-A*74:01. Among these, only the C-terminal 9-mer MVHQAISPR (positions 3–11 of GR11) was predicted as a binder and was therefore used as the putative binding core in the subsequent variant analyses.

#### 2.4.2 Proteasome cleavage

We assessed the cleavage of HIV proteins by the proteasome using NetChop 3.1 (https://services.healthtech.dtu.dk/services/NetChop-3.1/) (Nielsen et al., 2005). This server employs neural network predictions to identify cleavage sites by the human proteasome. We applied a threshold of 0.5 to define positive cleavage outcomes.

#### 2.4.3 Threshold sensitivity analysis

To assess the robustness of prediction classifications to the selected thresholds, we performed a sensitivity analysis using the continuous scores obtained in the original predictions. NetMHCpan results were reclassified using alternative %Rank thresholds of <0.25, <0.50, <0.75, and <1.00. NetChop predictions were independently reclassified using cleavage-score thresholds of ≥0.25, ≥0.50, and ≥0.75. We additionally evaluated the combined classification of HLA binding and predicted proteasomal cleavage under these alternative threshold combinations. No prediction was rerun; the sensitivity analysis consisted of reclassification of the original continuous prediction outputs. Detailed results are provided in **Supplementary Material 15**.

#### 2.4.4 Combined prediction for antigen processing pathway

For a comprehensive prediction encompassing TAP translocation, proteasome cleavage, and MHC binding, we turned to the IEDB Epitope Prediction and Analysis Tools (http://tools.iedb.org/processing). This tool provided us with specific processing ranking scores (Tenzer et al., 2005). To evaluate the impact of mutations on prediction accuracy, we considered the ranking position of the wild-type epitope. The parameters considered included HLA allele, epitope length, and immunoproteasome components.

Processing ranks were interpreted as relative ordinal measures within each prediction set rather than as quantitative effect estimates. Because the absolute processing scores may depend on the number and length of peptides evaluated in a given prediction, comparisons were based primarily on the relative position of each subtype-specific variant with respect to the corresponding reference epitope. Small differences among top-ranked peptides were not considered biologically meaningful in isolation. Instead, rank changes were interpreted together with HLA-binding and proteasomal-cleavage predictions as part of an integrated assessment of antigen processing and presentation.

#### 2.4.5 Criteria for geotargeted candidate prioritization

For the purposes of this study, a favorable geotargeted candidate was defined as an epitope previously associated with HIV control whose corresponding sequence in a locally circulating HIV subtype preserved the predicted molecular requirements for antigen processing and presentation. Candidate variants were evaluated for maintenance of HLA binding according to the NetMHCpan 4.1 thresholds, preservation of predicted C-terminal proteasomal cleavage according to NetChop 3.1, and preservation of the integrated antigen-processing profile generated by the IEDB processing tool. Because no universal positivity threshold is defined for the integrated processing score, changes in this parameter were assessed comparatively by examining the ranking position of each subtype-specific variant relative to the corresponding reference epitope. Mutations within the epitope or its flanking regions associated with loss of HLA binding, loss of predicted proteasomal cleavage, or a pronounced deterioration in the integrated processing ranking were interpreted as unfavorable for candidate prioritization. The “Overall assessment” reported in **Table 1** represents a qualitative synthesis of these criteria, integrating predicted HLA binding, proteasomal cleavage, relative processing rank, sequence robustness across viral subtypes, and the corresponding geographic HLA–subtype context. These categories were used for comparative prioritization and should not be interpreted as formally calibrated evidence grades or quantitative probabilities of vaccine efficacy.

**Table 1:** Geotargeting-based prioritization of HIV epitopes according to HLA context, circulating subtypes, and predicted antigen processing *Integrated interpretation of molecular predictions and regional host–pathogen context; priority settings are illustrative rather than universal recommendations*.

Epitope	HLA restriction(s)	Overall assessment	Geographic prioritization	Molecular rationale/main caveat
TW10	HLA-B*57:01, B*57:03, B*58:01	Strong candidate	Prioritize: Kenya, Uganda, South Africa, Zimbabwe. Deprioritize: Morocco, Mali. Insufficient data: Central African Republic (11_cpx not evaluated).	All tested A/B/C/D/CRF02_AG variants retained HLA binding, proteasomal cleavage, and a favorable combined processing profile. Low local HLA frequency lowers geographic priority only.
TL9	HLA-B*42:01; predicted B*51:01, B*07:02, B*08:01, B*39:01	Strong candidate	Prioritize: Zambia, Mali, Ghana. Deprioritize: Morocco, Libya. Caution: clade-B Q3T when B*08:01 is relevant.	Variants generally retained binding, cleavage, and favorable processing; the main exception was clade-B Q3T for B*08:01.
KF11	HLA-B*57:01, B*57:03; predicted B*58:01	Conditional candidate	Prioritize: Kenya, South Africa, Zimbabwe, Zambia, and selected CRF02_AG settings with adequate HLA representation. Avoid subtype-B F11V. Lower priority: Mali. Insufficient data: Central African Republic.	F11V abolished predicted binding and shifted relative processing rank from 1st to 29th; A/C/CRF02_AG variants remained favorable. No subtype-D sequence was available.
KK10	HLA-B*27:05	High-risk/sequence-dependent	No unconditional geographic recommendation. Avoid settings dominated by disruptive R2 escape variants; Morocco and Mali are low-priority contexts.	Several subtype-B variants lost predicted binding; proteasomal cleavage was affected in multiple viral backgrounds. HLA frequency alone is insufficient for prioritization.
AW11	HLA-B*44:03; predicted B*57:01, B*57:03, B*58:01	Validation-only/cleavage-limited	Potential validation settings: Kenya, South Africa, Zimbabwe, Uganda, Libya. Do not prioritize before cleavage validation. Lower priority: Mali. Insufficient data: São Tomé.	Binding and combined processing were generally favorable, but predicted proteasomal cleavage was inadequate across all tested subtypes; some subtype-B variants also lost binding.
GR11	HLA-A*74:01 (predicted 9-mer core MVHQAISPR)	Weak/subtype-dependent	No robust geographic recommendation. Do not prioritize clade A. CRF02_AG remains unresolved because of sequence-quality limitations; Morocco and Sudan are constrained settings.	Clade-A variants were predicted non-binders, cleavage was impaired in several backgrounds, and exploratory CRF02_AG outputs were excluded from biological interpretation.
TI8	HLA-B*51:01 only	Negative model for a generic vaccine	No broad recommendation. Avoid South Africa (clade C), Mali/Ghana (CRF02_AG), and other A/C/D/CRF02_AG settings carrying I8 substitutions. Subtype B requires sequence-level confirmation.	I8 substitutions caused loss of HLA-B*51:01 binding, marked deterioration in relative processing rank, and, for several variants, loss of proteasomal cleavage. Single-HLA restriction further limits reach.

Geographic prioritization integrates available HLA frequencies, circulating HIV subtype(s), HLA-binding predictions, proteasomal cleavage, and combined processing. No formal combined population-coverage percentage was calculated. Any summed HLA-frequency values used in the extended analysis are descriptive only and must not be interpreted as individual-level coverage because HLA co-inheritance, linkage disequilibrium, and haplotype structure are not captured. Overall assessments are qualitative syntheses of computational evidence, not calibrated evidence grades or probabilities of vaccine efficacy.

## 3 Results

### 3.1 Maps and HLA allele frequencies

The maps produced by our study revealed relationships between HLA allele prevalence and the presence of HIV subtypes in different geographical areas. Notably, the HLA-A*74:01 and HLA-B*58:01 alleles were consistently identified in regions with a high incidence of HIV-1 subtypes A and CRF02_AG, as detailed in **Supplementary Materials 1** and **2**, respectively.

The prevalence values of HLA-B*27:05 and HLA-B*57:01 were noteworthy in specific African countries, as highlighted in **Supplementary Materials 3** and **4**, respectively. These prevalence patterns aligned with the dominant HIV-1 subtype C in Southern Africa, which reaches nearly 98.8% (Hemelaar et al., 2019). Furthermore, HLA-B*57:03 demonstrated elevated prevalence rates in Zambia (5.7%) and Zimbabwe (4.4%). Remarkably, these are regions where HIV-1 subtype C was also highly prevalent, as indicated in **Supplementary Material 5**.

Notably, HLA-B*44:03 had substantially high frequency in various African countries, as documented in **Supplementary Material 6**. Importantly, these countries share a commonality in the prevalence of HIV subtypes A, B, C and CRF02_AG. Conversely, HLA-B*42:01 showed prevalence patterns aligned with the dominance of HIV subtypes C, CRF02_AG and CRF06-cpx, as outlined in **Supplementary Material 7**. The full picture from the available information about the allele frequencies in each country is summarized in **Table 2**.

**Table 2:** Frequencies of HLA alleles associated with HIV control across the analyzed African countries, as recovered from the Allele Frequency Net Database, together with the HIV-1 subtype(s) considered for each country.

Country	HLA Alleles frequencies (%)							HIV subtypes
	B*27:05	B*58:01	A*74:01	B*57:03	B*57:01	B*42:01	B*44:03	
Burkina Faso	NI	5.39	3.34	NI	NI	7.72	NI	06_cpx, 02_AG
Cameroon	0.30	5.74	6.28	3.14	NI	3.60	8.81	02_AG, A
Cape Verde	2.00	3.60	1.20	5.60	1.60	2.40	2.80	G, 02_AG
Central African Republic	NI	2.78	NI	0.00	NI	1.51	7.26	11_cpx, A
Ghana	NI	4.20	5.34	0.38	0.38	11.07	8.78	02_AG
Guinea Bissau	0.00	7.80	7.05	0.80	0.00	1.50	3.10	02_AG
Ivory Coast	NI	2.30	NI	4.60	NI	3.40	3.40	02_AG
Kenya	0.22	8.22	6.17	2.59	1.86	7.87	1.46	A
Libya	NI	3.40	NI	3.00	0.90	0.40	5.10	02_AG
Mali	0.00	2.20	3.60	0.70	0.00	13.80	1.50	02_AG
Morocco	0.90	2.86	0.89	1.21	2.35	1.97	8.10	B
Rwanda	NI	NI	NI	6.90	NI	4.70	NI	A
Sao Tome Island	0.54	3.56	4.08	2.54	NI	2.56	1.01	NI
Senegal	NI	6.90	2.20	0.50	NI	0.50	0.50	02_AG
South Africa	2.10	4.99	2.34	1.78	3.09	5.15	5.08	C
Sudan	0.80	4.19	0.50	1.30	1.43	1.37	1.50	D,C
Tunisia	NI	3.17	1.05	1.95	3.00	1.30	2.10	B, 02_AG
Uganda	1.20	5.04	7.39	2.09	3.10	3.86	2.56	D,A
Zambia	0.00	0.00	2.30	5.70	1.10	14.80	2.30	C
Zimbabwe	NI	4.40	2.70	4.40	0.40	6.90	5.80	C

*NI, not informed.

### 3.2 Epitope mutations and HLA binding

In this step, we investigated the impact of mutations identified in epitopes associated with HIV control in the context of population genetics across African countries and the prevalent HIV strains circulating within these geographic regions.

Circulating African sequences for Subtypes A, B, C, D, and CRF02_AG variants showed no altered binding and proteasome predictions for the previously described HLAs (B*57:01, B*57:03, and B*58:01) within the TW10 epitope region. Additionally, variants from all clades remained among the highest-ranked peptides in the combined antigen-processing predictions. When considered together with the preserved HLA-binding and proteasomal-cleavage predictions, this supported maintenance of their overall predicted processing profile. All analyzed sequences showed negative predictions for binding to other investigated HLA alleles, even as weak binders. **Figure 2** shows an illustrated landscape of the impact of the mutations on the TW10 epitope. The complete predictions for all epitopes are detailed in **Supplementary Material 8**.

**Figure 2:**
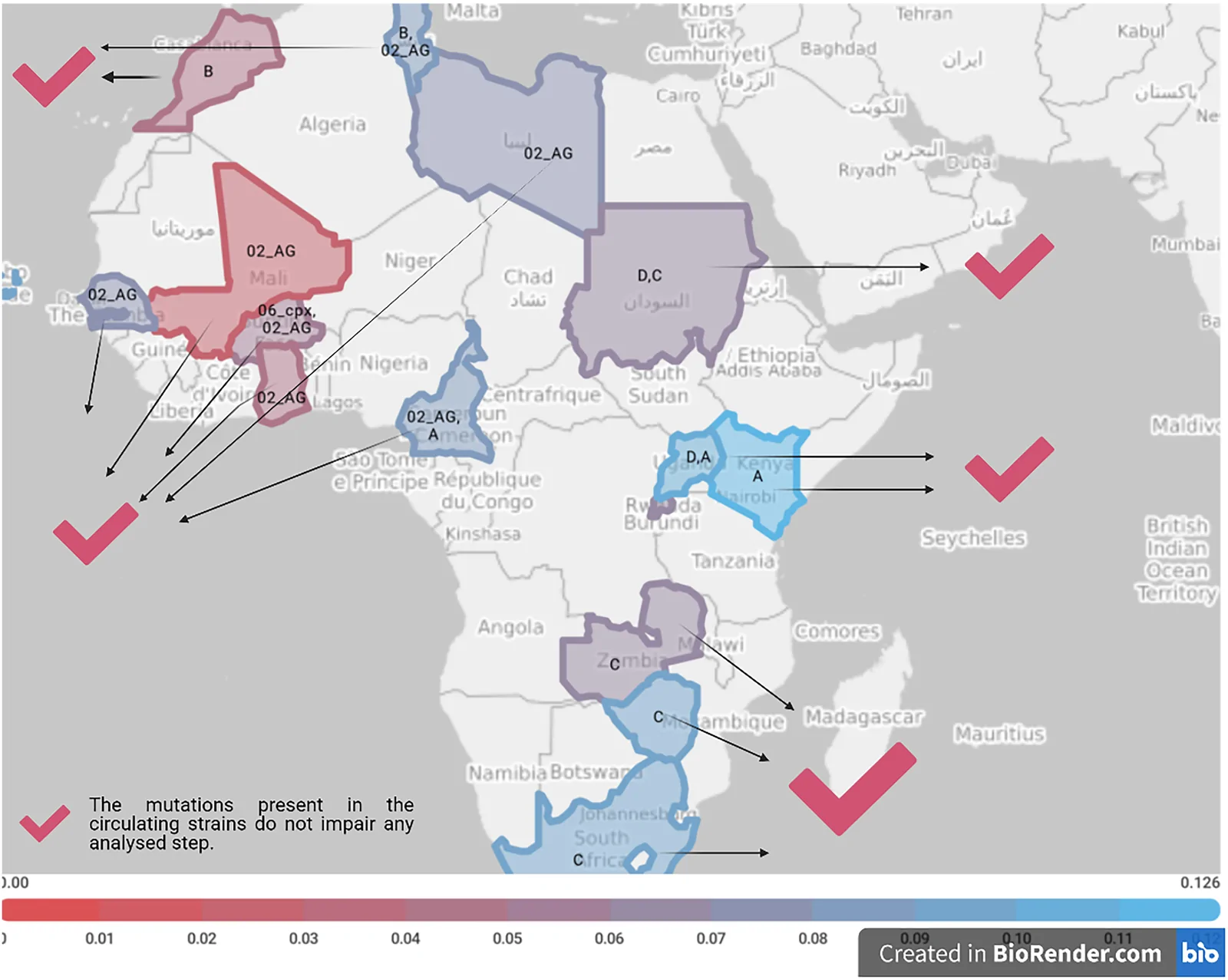
African map summarizing the predicted effects of subtype-specific TW10 variants in the analyzed geographic settings. Country colors indicate the combined frequency of HLA-B*57:01, HLA-B*57:03, and HLA-B*58:01 associated with TW10 presentation, while subtype labels indicate the HIV-1 lineages considered. Check marks indicate settings in which the analyzed TW10 variants preserved predicted HLA binding, proteasomal cleavage, and the overall antigen-processing profile.

The TL9 epitope within the p24 Gag protein was originally described for HLA-B*42:01 (Goulder and Walker, 2012). Our predictions suggested that this epitope could also be presented by other HLA alleles, including B*51:01, B*07:02, B*08:01, and B*39:01. All recovered variants retained binding capability and remained among the highest-ranked peptides in the combined processing prediction. Minor differences among the top-ranked positions were not interpreted as meaningful changes in processing. The only exception was the Clade B Q3T mutation, which was predicted to be a non-binder for B*08:01. Proteasome cleavage predictions indicated adequate processing for all sequences considered (**Supplementary Material 9**).

The TI8 epitope from the reverse transcriptase (RT) protein (Clade B) is specifically restricted to HLA-B*51:01, with no predictions for other analyzed alleles. The wild-type epitope achieved the 10th position in the combined processing prediction. This HIV target presented several issues in its variants. Some mutated Clade B epitope sequences were also predicted as strong binders for HLA-B*51:01, but the I8T and I8R mutations resulted in a loss of binding affinity, ranking 97th and 40th position in the combined prediction, respectively. Similarly, I8T, I8R and I8K mutations in Clade C sequences were predicted as non-binders and ranked 91st, 60th, and 32nd, respectively. For Clade A sequences, the I8T, I8R, and I5L/I8T mutations were not predicted to be binders for HLA-B*51:01, ranking 93rd, 32nd, and 98th positions in the predictions for proteasome, TAP, and MHC binding. In Clade D sequences, the I8T mutation was not predicted as a binder for HLA-B*51:01 and was ranked 99th in the combined processing prediction. For the CRF02_AG subtype, the I8K, I8T, and I8R mutations were not binders and were ranked 63rd, 96th and 31st, respectively. Independent predictions for proteasome cleavage sites were abolished for sequences from subtypes B (mutation I8T), C (mutation I8T), A (mutation I5L/I8T), D (mutation I8T), and CRF02_AG (mutation I8T). **Figure 3** summarizes the impact of these mutations on the TI8 epitope. These pronounced rank deteriorations were interpreted as relative changes in processing priority rather than as quantitative effect sizes and were considered together with the concomitant loss of HLA binding and, for several variants, impaired proteasomal cleavage.

**Figure 3:**
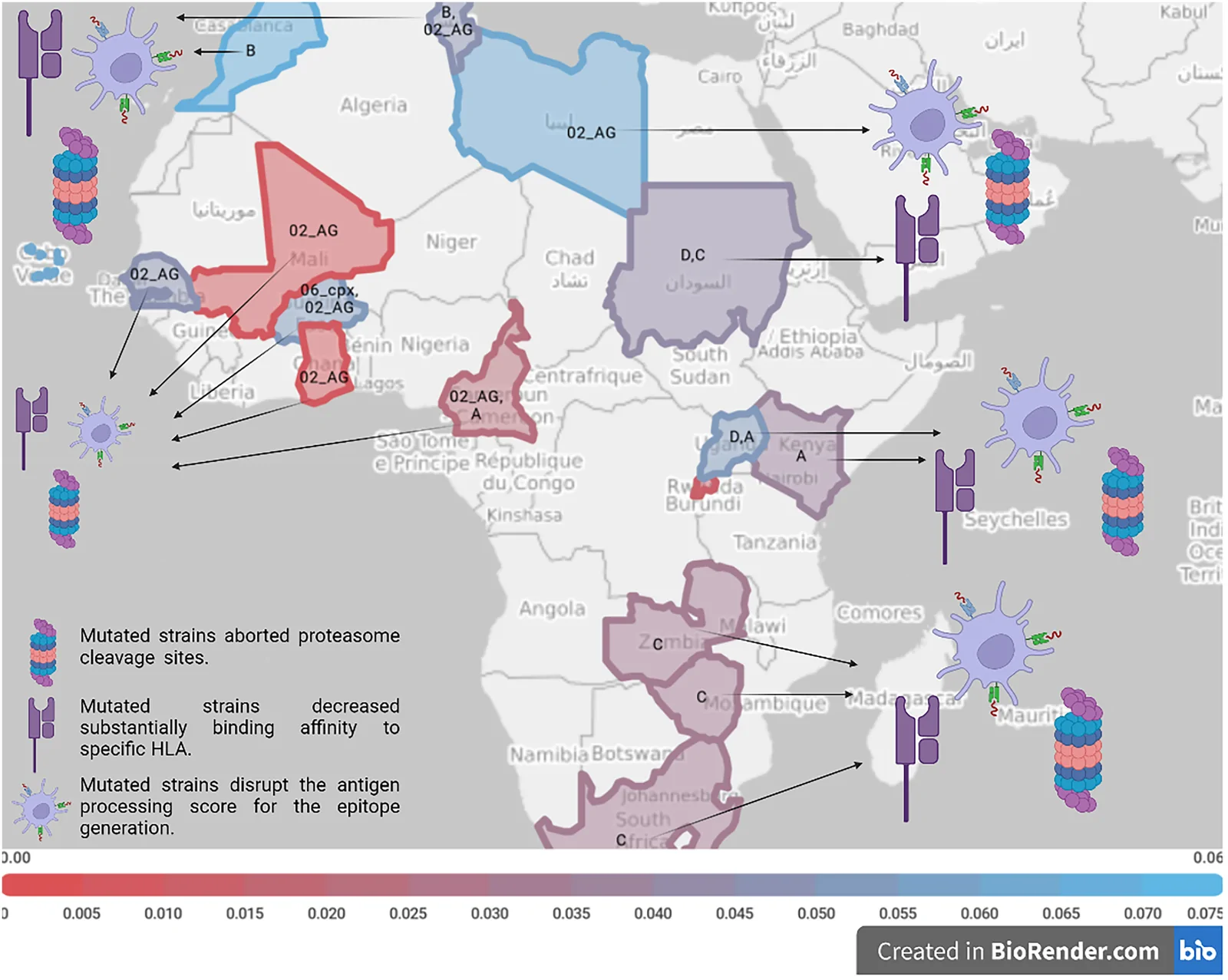
African map summarizing the predicted effects of subtype-specific TI8 variants in the analyzed geographic settings. Country colors indicate the frequency of HLA-B*51:01, the HLA restriction associated with TI8, while subtype labels indicate the HIV-1 lineages considered. Symbols indicate subtype-specific variants predicted to abolish proteasomal cleavage, reduce HLA-B*51:01 binding, and/or impair the integrated antigen-processing profile.

The KF11 epitope, located in the p24 Gag protein, was originally restricted to HLAs B*57:01 and B*57:03. Our analysis revealed that KF11 peptide variants were also predicted to be strong or weak binders for the HLA-B*58:01 allele.

The F11V mutation in subtype B was associated with a marked deterioration in the relative processing ranking for HLA-B*57:01, moving from the first to the 29th position. This change was interpreted together with the concomitant loss of predicted HLA binding rather than as an independent quantitative measure of effect. Mutations in subtype A, C and CRF02_AG sequences maintained both the binding affinity for HLAs B*57:01 and B*57:03, as well as their processing scores, placing them at the top of the ranking. We did not find any KF11 sequences for subtype D in Uniprot. Proteasome cleavage was maintained for all sequences from subtypes B, C, A and CRF02_AG (**Supplementary Material 10**).

The KK10 Epitope, p24 Gag protein is restricted to HLA-B*27:05 (Clade B). However, mutations in subtype B sequences (R2Q/L6M, R2K/L6M, R2K/L6I, R2G, R2T) resulted in a loss of its binding capacity and compromised its ability to undergo correct processing, particularly for R2G and R2T. The same occurred for the Clade C R2K/L6M mutation, which was predicted as a non-binder and showed a lower relative processing position (8th). In contrast, the I5V variant retained its strong-binding status and remained among the highest-ranked peptides in the combined processing prediction. Likewise, Clade A and CRF02_AG variants maintained their binding status and remained top-ranked in the processing analysis. The prediction of proteasome cleavage to generate the correct peptide was impacted in sequences from subtypes B, C, A, D, and CRF02_AG (**Supplementary Material 11**).

Another epitope from the p24 Gag protein, AW11, is specifically restricted to HLA-B*44:03 (Clade C). Our analysis suggests that the epitope could also bind to HLA-B*57:01, B*57:03 and B*58:01. The T5S/D7E/W11R and E2G/T5S/D7E mutations in subtype B abolished predicted HLA binding and were accompanied by marked deterioration in the relative processing ranking, reaching the 19th and 26th positions, respectively. In contrast, Clade A, C, D, and CRF02_AG sequences retained strong predicted binding and remained top-ranked in the combined processing analysis. However, proteasomal cleavage was predicted to be inadequate for sequences from all tested subtypes, underscoring the significance of alterations in the surrounding regions of the epitope sequence (**Supplementary Material 12**).

The final epitope derived from the p24 Gag protein, GR11, is noteworthy as it is the only epitope originally associated with the locus A (HLA-A*74:01) in this work. Since HLA-A74:01 typically presents nonamers, the three possible overlapping 9-mer peptides derived from GR11 were evaluated for binding. Only the C-terminal 9-mer MVHQAISPR was predicted as a binder and was therefore used as the putative HLA-A74:01-binding core for the subsequent subtype-specific analyses. In Clade B, all described mutations in the epitope region did not impact its binding to the described allele or its overall processing capacity. Sequences from Clade A were predicted as non-binders for HLA-A*74:01 and showed unfavorable relative processing rankings. For CRF02_AG, candidate sequences were retrieved and exploratory prediction outputs were generated; however, the underlying records were flagged during sequence-quality control and were not considered sufficiently reliable for biological interpretation. These CRF02_AG outputs were therefore treated as missing data rather than as negative predictions. Across the evaluable subtype-specific sequences, predicted proteasomal cleavage was also lost in several backgrounds (B, C, A, and D), as depicted in **Supplementary Material 13**. Accordingly, geographic settings dependent on these unresolved CRF02_AG records were not used to support a definitive GR11 recommendation, as depicted in **Supplementary Material 13**.

Threshold sensitivity analysis showed that NetMHCpan classifications were highly stable around the default %Rank cutoff, with only minor changes when more stringent or permissive thresholds were applied. In contrast, NetChop predictions showed greater threshold dependence, which was concentrated in a subset of epitopes. Several epitopes maintained the same cleavage interpretation across all evaluated thresholds, whereas others showed greater variation when more permissive or stringent cleavage criteria were applied (**Supplementary Material 15**).

## 4 Discussion

In this study we investigated variations in epitope regions among different HIV subtypes and their impact on antigen processing and presentation, particularly in individuals with HLA alleles associated with EC. Using Africa as a focal point due to its likely role in the origins of the global HIV pandemic and the persistent burden of AIDS across African countries, we show how variations in allele frequencies and viral diversity could hinder the efficacy and coverage of vaccines that disregard these factors.

We started our analysis by reviewing various HLAs alleles previously associated with the modulation of cellular immune response to HIV. Although a primary association between some HIV epitopes and particular HLA alleles has been reported (Goulder and Walker, 2012), the relevance of this connection for vaccine design seems highly dependent on HIV subtypes and HLA allele frequencies in the host population. For example, in African-American patients with HIV subtype B, certain HLAs such as HLA-B*14, HLA-B*57:01, and HLA-B*57:03 were associated with better viral control, while other HLAs, including HLA-B*15:10 and HLA-B*35:01, were associated with poorer outcomes (Lazaryan et al., 2011). Other HIV studies have demonstrated that a single amino acid difference between HLA subtypes, such as HLA-B*42:01 and HLA-B*42:02, is sufficient to exert a significant impact on disease outcomes (Kløverpris et al., 2012). Similarly, HLA-B*58:02 has been linked to faster disease progression, whereas HLA-B*58:01 is associated with slower disease progression (Ngumbela et al., 2008).

From this perspective, we wanted to closely examine epitope targets implicated in viral clearance to achieve a deeper understanding of HLA alleles associated with elite control (Goulder and Walker, 2012), and explain why - and where - certain alleles confer resistance/susceptibility to specific HIV subtypes. To this end, we selected epitopes presented by those alleles commonly associated with elite control. Importantly, the use of EC-associated HLA–epitope pairs as candidate targets does not imply that these epitopes would reproduce the elite-controller phenotype after vaccination, which is shaped by multiple host and immune factors; rather, they provide biologically supported targets for evaluating how host–virus diversity may affect antigen processing and presentation.

In this sense, we predicted that particular epitopes, such as TW10, maintain strong binding affinity and high processing rank despite common mutations. TW10 is a promising target for vaccine or immunotherapy development, as it remains a robust binder and ranks highly for processing steps across multiple HLA alleles, including B*57:01, B*57:03 and B*58:01. Given the prevalence of these HLAs in countries such as South Africa, Zimbabwe and Rwanda, these regions could serve as suitable locations for testing vaccines with this particular epitope. Likewise, the TL9 epitope demonstrates potential binding to HLA-B*42:01, with additional binding predictions for other HLA alleles such as B*07:02, B*08:01, B*39:01, and B*51:01 across all tested clades, along with favorable processing pathway ranking predictions. This suggests potential suitability for testing in Zambia, Mali and Ghana, where several of these HLA alleles are represented in the population.

Another example of potential candidates for vaccine approaches includes the KF11 epitope, which retains strong binding affinity and high processing rank in Clades B, C, A and CFR02_AG HIV sequences. It exhibits a notable affinity for HLA-B*57:01, B*57:03, and B*58:01. Suitable regions for the use of this epitope include South Africa, Zimbabwe, Zambia and Kenya. Similarly, the AW11 epitope originally predicted for HLA-B*44:03, shows promising potential for HLA-B*57:01, B*57:03 and B*58:01 in all clades tested. Regions such as Libya, South Africa, Zimbabwe, Uganda and Kenya would be ideal for testing this epitope.

In contrast, the TI8 epitope has mutations in the Clade C and A sequences that affect HLA binding prediction even for HLA-B*51:01. In this sense, it should not be used in South Africa, for example, where the prevalence of HIV Clade C is 98% and the prevalence of B*51:01 is 3%. This epitope also presents impaired proteasome cleavage prediction and antigen processing scores in some analyzed sequences. Considering these factors, TI8 may serve as a negative model for a generic epitope-based HIV vaccine, expected to have limited efficacy and coverage across the African continent.

Another epitope that requires a personalized approach for target prediction and application is Gag KK10, one of the first HIV T cell targets to be associated with slow disease progression. The predominant escape mutation of this epitope is R264K, which occurs at HLA anchor position-2, and disrupts the binding and presentation by HLA-B*27. The occurrence of this mutation is associated with a higher risk of developing AIDS in HLA-B*27+ individuals (Kløverpris et al., 2016). In this study, we predicted that the R264K mutation also has an impact on other steps of the antigen presentation pathway. Moreover, the mutations observed in this epitope diminish its binding strength and processing rank for Clade B, while maintaining it for Clades C, A, D, and CRF02_AG. Countries such as South Africa, Zimbabwe, and Zambia would be suitable for the use of this epitope, whereas it would not be effective in Morocco.

The examples above illustrate a central challenge in HIV vaccine development: epitope performance is likely to depend on the interaction between local HLA allele distributions, circulating HIV subtypes, and subtype-specific escape mutations affecting peptide-presentation. These factors may help explain the limited protection observed in previous immunotherapeutic interventions. Using predictive tools, we identified geographic settings in which selected epitopes may retain favorable binding and processing profiles, as well as settings where their use may be less appropriate (**Table 1**).

Importantly, no single genetic variant identified to date confers the EC state uniformly in all populations, and the combined effects of multiple genetic variants are likely required for effective HIV-1 control (Crux and Elahi, 2017). Consistent with this view, a computational study evaluating the additive effects of HLA alleles on HIV infection suggested that broader CD8+ T-cell targeting across HLA alleles, rather than targeting associated with either allele alone, may contribute to this additive effect (Matthews et al., 2012). These findings support the inclusion of multiple CD8+ T-cell targets in cellular vaccine design. However, the purpose of the present geotargeting framework is not to define a universal multi-epitope formulation, but to inform the selection of candidate targets according to the local distribution of HIV variants, HLA alleles, and predicted antigen-processing constraints. In a translational setting, combinations of epitopes could subsequently be optimized at national, regional, or subpopulation levels using this information, together with individual-level HLA haplotype, linkage-disequilibrium, and immunodominance data. Note that certain HLA alleles are more limited to African populations (e.g., HLA-A*74:01, B*15:03, B*58:02) (Tshabalala et al., 2015), and epitope targets restricted to these populations might therefore be less applicable to immunotherapeutic interventions in locations where these alleles are rare.

A limitation concerns uncertainty in the HLA allele-frequency data used for geographic prioritization. The available datasets vary substantially in sample size and population ancestry composition, and the sample-size-weighted country-level estimates should therefore not be interpreted as nationally representative allele frequencies. To assess the dependence of these estimates on the composition of the available samples, we performed a leave-one-population-out sensitivity analysis for country–allele combinations represented by multiple population samples, sequentially recalculating the weighted frequency after exclusion of each contributing sample. The resulting ranges are provided in **Supplementary Material 14** and offer a descriptive measure of estimate robustness. Nevertheless, this analysis does not account for population structure or other sources of sampling bias, and estimates derived from a single available population sample remain particularly uncertain. A related source of uncertainty concerns the geographic representation of HIV subtypes. Sequence frequencies in the Los Alamos HIV Sequence Database reflect available deposited sequences and are influenced by heterogeneous sequencing intensity across countries and studies; they should therefore not be interpreted as direct estimates of subtype prevalence. Although major subtype patterns were compared with independent molecular epidemiological data, uneven viral sequence sampling remains a limitation of the present proof-of-concept and should be addressed in future implementations using contemporary population-based molecular surveillance data whenever available. An additional point is its reliance on immunoinformatics methods to predict how HIV sequence diversity may affect epitope processing and presentation under specific HLA restrictions. Importantly, the present framework evaluates the likelihood of antigen generation and HLA presentation, but does not directly predict downstream T-cell recognition or immunogenicity. At the same time, this limitation reflects a broader issue in epitope-based vaccine design: candidate selection often relies heavily, and sometimes almost exclusively, on HLA binding predictions. Although binding affinity is necessary for peptide presentation, it is not sufficient to predict whether an epitope will become an effective T-cell target. Protective responses are shaped by multiple steps of the antigen presentation pathway, including proteasomal cleavage, peptide transport, HLA loading, peptide-HLA stability at the cell surface, antigen abundance, and the capacity of the peptide-HLA complex to sustain productive T-cell recognition. These factors contribute to immunodominance, which is ultimately more relevant to vaccine efficacy than binding affinity alone. Similarly, the processing rankings used here should be interpreted as relative prioritization measures rather than quantitative effect sizes or direct measures of T-cell immunodominance. Small differences among highly ranked peptides were therefore not considered biologically meaningful in isolation, whereas larger rank deteriorations were interpreted only when supported by concordant changes in HLA binding, proteasomal cleavage, or both. Current computational tools remain uneven across these steps. Recent advances in deep learning, protein language models, AlphaFold-based peptide–MHC structural modeling, and immunopeptidomics are beginning to extend computational vaccine design beyond conventional sequence-based HLA-binding prediction. However, the generalization of these approaches to novel peptide–HLA contexts and genetically diverse populations remains an important challenge (Gfeller et al., 2023; Mikhaylov et al., 2024; Villanueva-Flores et al., 2025). HLA binding predictors are generally more mature than models of antigen processing, peptide-HLA stability, and immunogenicity (Reynisson et al., 2020; Gfeller et al., 2023; Turner et al., 2023), but relying on binding alone can lead to the prioritization of epitopes that are predicted to bind well yet may be unable to trigger dominant T-cell responses. This gap may help explain why some epitope-based vaccines and T-cell-based immunotherapies have not achieved their expected efficacy despite rational target selection. As an external consistency check, we compared representative exact subtype-specific variants analyzed here with previously published experimental data. Several predicted HLA-binding effects, including loss-of-binding variants of KK10 and TI8 as well as variants predicted to retain presentation, were concordant with independent peptide–HLA, structural, and/or T-cell functional evidence (**Supplementary Material 16**). These observations provide experimental support for representative predictions generated by the framework, while not replacing prospective validation of variants for which direct experimental evidence is unavailable. Therefore, the limited accuracy of current predictors for antigen processing, stability, and immunogenicity should not justify excluding these steps from vaccine design. Rather, it emphasizes the need to improve and integrate these models, particularly using datasets that better represent genetically diverse and underrepresented populations. Future geotargeting approaches should therefore combine HLA binding prediction with antigen processing and immunogenicity/immunodominance prediction models, peptide-HLA stability estimates, structural information, immunopeptidomics, and T-cell repertoire data to identify regionally relevant epitope combinations that are more likely to be processed, presented, and capable of inducing immunodominant T-cell responses associated with durable HIV control.

Translation of population-adapted vaccine designs would also require consideration of regulatory, manufacturing, supply-chain, and cost constraints. Although modular platforms such as mRNA vaccines may facilitate adaptation of antigenic content, their implementation would still need to account for production scale, storage and delivery requirements, and equitable access, particularly in resource-limited settings.

In this study, we integrated immunoinformatics methods for the prediction of proteasomal cleavage, TAP transportation, HLA binding, and overall processing ranking to evaluate epitope generation across HIV variants and host populations with different HLA allele distributions. Our findings support the rationale for incorporating regional host and viral diversity into HIV vaccine-target selection and provide a computational proof-of-concept for geographically informed prioritization. By integrating these host–pathogen variables, the framework may help identify context-specific epitope targets for subsequent experimental evaluation and refinement. However, the specific epitope and geographic predictions generated here require prospective experimental validation before conclusions regarding vaccine efficacy or translational utility can be established. More broadly, this framework may also inform future target-selection strategies for other highly variable viruses.

## Data Availability

The original contributions presented in the study are included in the article/**Supplementary Material**. Further inquiries can be directed to the corresponding author.
