# Supplementary material for: Upgrading the HIV vaccine paradigm: accounting for host-pathogen diversity through geotargeting-based vaccine design
Source: Front Cell Infect Microbiol. 2026 Oct 16;16:1927865. doi: 10.3389/fcimb.2026.1927865 (PMC13719621; doi:10.3389/fcimb.2026.1927865)
Supplement: Supplementary file 1 [file Table1.docx]

Supplementary material 1.png

African Continent Map showing the prevalence of HLA-A*74:01. The color scale below the map indicates the allele frequency in each country.

Supplementary material 2.png

African Continent Map showing the prevalence of HLA-B*58:01.The color scale below the map indicates the allele frequency in each country.

Supplementary material 3.png

African Continent Map showing the prevalence of HLA-B*27:05. The color scale below the map indicates the allele frequency in each country.

Supplementary material 4.png

African Continent Map showing the prevalence of HLA-B*57:01. The color scale below the map indicates the allele frequency in each country.

Supplementary material 5.png

African Continent Map showing the prevalence of HLA-B*57:03. The color scale below the map indicates the allele frequency in each country.

Supplementary material 6.png

African Continent Map showing the prevalence of HLA-B*44:03. The color scale below the map indicates the allele frequency in each country.

Supplementary material 7.png

African Continent Map showing the prevalence of HLA-B*42:01. The color scale below the map indicates the allele frequency in each country.

Supplementary material 8.xlsm

Compiled subtype-specific prediction outputs for the analyzed HIV epitopes (TW10, KF11, AW11, TL9, KK10, TI8, and GR11), including epitope sequences and mutations, UniProt accessions, proteasomal-cleavage predictions, HLA-binding predictions, and integrated antigen-processing rankings.

Supplementary material 9.jpeg

African Continent Map showing the main effects associated with the TL9 Epitope mutations present in the circulating subtype for each specific location. The colors in the countries indicate the combined frequency of the HLA-B*42:01, B*07:02, B*08:01, B*39:01 alleles associated with the corresponding epitope.

Supplementary material 10.jpeg

African Continent Map showing the main effects associated with the KF11 Epitope mutations present in the circulating subtype for each specific location. The colors in the countries indicate the combined frequency of the HLA-B*57:01, B*57:03 and B*58:01 alleles associated with the corresponding epitope.

Supplementary material 11.jpeg

African Continent Map showing the main effects associated with the KK10 Epitope mutations present in the circulating subtype for each specific location. The colors in the countries indicate the frequency of the HLA-B*27:05 allele associated with the corresponding epitope.

Supplementary material 12.jpeg

African Continent Map showing the main effects associated with the AW11 Epitope mutations present in the circulating subtype for each specific location. The colors in the countries indicate the combined frequency of the HLA-B*44:03, B*57:01, B*57:03 and B*58:01 alleles associated with the corresponding epitope.

Supplementary material 13.png

African Continent Map showing the main effects associated with the GR11 Epitope mutations present in the circulating subtype for each specific location. The colors in the countries indicate the frequency of the HLA-A*74:01 allele associated with the corresponding epitope. An asterisk (*) indicates incomplete data due to insufficient sequence quality for reliable interpretation of the full antigen-processing analysis; these settings were treated as missing data rather than as unfavorable predictions.

Supplementary material 14.xlsx

Source-level HLA allele-frequency data and quality-control calculations used to derive the sample-size-weighted country estimates, including contributing population samples and sample sizes, consolidation of duplicate-resolution records, and leave-one-population-out sensitivity analyses.

Supplementary material 15.xlsx

Threshold-sensitivity analysis of NetMHCpan and NetChop predictions using alternative HLA-binding and proteasomal-cleavage cutoffs. The analysis reclassifies the continuous prediction outputs generated in the original analyses without rerunning the prediction tools.

Supplementary material 16.xlsm

External experimental consistency analysis for representative subtype-specific HIV epitope variants evaluated in this study, comparing the computational predictions with published peptide-HLA binding, structural, tetramer, and/or T-cell functional evidence.
